# The risk and safety profile of electronic nicotine delivery systems (ENDS): An umbrella review

**DOI:** 10.1093/eurpub/ckac129.638

**Published:** 2022-10-25

**Authors:** T Asfar, W Maziak

**Affiliations:** 1 Public Health Sciences, University of Miami, Miller School of Medicine, Coral Gables, USA; 2 Department of Epidemiology, Florida International University, Miami, USA

## Abstract

**Background:**

Balancing electronic nicotine delivery systems (ENDS) health communication between limiting the harm for new users while offering current smokers the benefits of reduced-risk products is challenging. This umbrella review aims to summarize the evidence about ENDS risk and safety health profile to inform ENDS health communication strategies.

**Methods:**

Six databases were searched for systematic reviews presenting evidence on ENDS health effects. Ninety reviews divided into 5 categories were included: toxicity = 20, health effects = 40, smoking cessation = 24, transition to combustible cigarettes (CCs) = 13, and industry claims = 4. Findings were summarized narratively. Meta-analyses were conducted when appropriate. Quality assessment was conducted using the Measurement Tool to Assess Systematic Reviews. The Institute of Medicine's Levels of Evidence Framework was used to classify the evidence into high-level, moderate, limited-suggestive, and limited-not-conclusive.

**Results:**

We found high-level evidence that ENDS exposes users to toxic substances; increases the risk of asthma; leads to nicotine dependence; causes serious injuries due to explosion or poisoning; increases smoking cessation in clinical trials but not in observational studies; increases CCs initiation; and exposure to ENDS marketing increases its use/intention-to-use. Evidence was moderate for ENDS association with mental health and substance use, and limited-not-conclusive for its association with cardiovascular, cancer, ear, ocular, and oral diseases, and pregnancy outcomes.

**Conclusions:**

Currently, ENDS communication can focus on high-level evidence related to ENDS association with toxicity, nicotine addiction, respiratory disease, ENDS-specific harm (explosion, poisoning), and anti-ENDS industry sentiment. Further research is needed to assess the full spectrum of ENDS health profiles.

**Key messages:**

• Further research is needed to establish the risk and safety health profile of ENDS with consideration of the wide variety of ENDS, their frequency of use, and different segments of the population.

• More prospective cohort studies that take place over longer time periods and with larger sample sizes are necessary to gauge accurately the short- and long-term health-related risks of ENDS.

